# STN1 OB Fold Mutation Alters DNA Binding and Affects Selective Aspects of CST Function

**DOI:** 10.1371/journal.pgen.1006342

**Published:** 2016-09-30

**Authors:** Anukana Bhattacharjee, Jason Stewart, Mary Chaiken, Carolyn M. Price

**Affiliations:** 1 Department of Cancer Biology, University of Cincinnati, Cincinnati, Ohio, United States of America; 2 Department of Biological Sciences, University of South Carolina, Columbia, South Carolina, United States of America; Masaruk University, CZECH REPUBLIC

## Abstract

Mammalian CST (CTC1-STN1-TEN1) participates in multiple aspects of telomere replication and genome-wide recovery from replication stress. CST resembles Replication Protein A (RPA) in that it binds ssDNA and STN1 and TEN1 are structurally similar to RPA2 and RPA3. Conservation between CTC1 and RPA1 is less apparent. Currently the mechanism underlying CST action is largely unknown. Here we address CST mechanism by using a DNA-binding mutant, (STN1 OB-fold mutant, STN1-OBM) to examine the relationship between DNA binding and CST function. *In vivo*, STN1-OBM affects resolution of endogenous replication stress and telomere duplex replication but telomeric C-strand fill-in and new origin firing after exogenous replication stress are unaffected. These selective effects indicate mechanistic differences in CST action during resolution of different replication problems. *In vitro* binding studies show that STN1 directly engages both short and long ssDNA oligonucleotides, however STN1-OBM preferentially destabilizes binding to short substrates. The finding that STN1-OBM affects binding to only certain substrates starts to explain the *in vivo* separation of function observed in STN1-OBM expressing cells. CST is expected to engage DNA substrates of varied length and structure as it acts to resolve different replication problems. Since STN1-OBM will alter CST binding to only some of these substrates, the mutant should affect resolution of only a subset of replication problems, as was observed in the STN1-OBM cells. The *in vitro* studies also provide insight into CST binding mechanism. Like RPA, CST likely contacts DNA via multiple OB folds. However, the importance of STN1 for binding short substrates indicates differences in the architecture of CST and RPA DNA-protein complexes. Based on our results, we propose a dynamic DNA binding model that provides a general mechanism for CST action at diverse forms of replication stress.

## Author Summary

Mammalian CST (CTC1/STN1/TEN1) is a three protein complex that aids in several steps during telomere replication and has genome-wide roles during recovery from replication fork stalling. Loss of CST leads to abnormalities in telomere structure, genomic instability and defects in chromosome segregation. Currently, we do not understand how CST acts to ensure the resolution of very diverse types of replication problem. We set out to address this question by studying a mutant form of CST that was predicted to alter DNA binding. The mutations are in the STN1 subunit. *In vivo*, the STN1 mutant (STN1-OBM) affects some aspects of CST function while others are normal. The effects of STN1-OBM do not align with the telomeric versus non-telomeric roles of CST but instead separate out different aspects of CST function at telomeres and genome-wide. *In vitro* binding studies indicate that STN1-OBM disrupts binding to only short DNA substrates. Since CST is likely to encounter DNA substrates of varied length and structure *in vivo* as it helps resolve different replication problems, this finding starts to explain why STN1-OBM affects only certain aspects of CST function. Our *in vitro* binding studies also shed light on how CST actually binds to DNA and they suggest a novel “dynamic binding model” that provides a mechanistic explanation for how CST helps resolve a diverse array of replication problems to preserve genome stability.

## Introduction

Although DNA replication must occur rapidly and with high fidelity, the replisome frequently encounters obstacles such as DNA damage or repetitive sequence that cause the replication fork to stall. Since stalled forks can lead to double strand breaks and genomic instability, multiple pathways exist to ensure their resolution [1,2]. Telomeres pose a particular challenge to the replication machinery due to their repetitive G-rich sequence and the inability of DNA polymerase to completely replicate the DNA 5’ terminus [3–5]. To ensure telomeres are duplicated efficiently, the replication process occurs in several distinct steps [3,6,7] and involves a number of ancillary proteins [8–11]. First, the repetitive dsDNA is duplicated by the replisome with assistance from various accessory factors. Next, the chromosome ends are processed by nucleases to form a single-stranded overhang on the 3’ G-rich strand (termed the G-overhang). In telomerase positive cells, the G-overhang is then extended by telomerase. Finally, much of the elongated overhang is converted to duplex DNA by DNA polymerase alpha (pol α) in a process known as C-strand fill-in. This leaves a short G-overhang that is then bound by telomere proteins.

CST is a protein complex that binds ssDNA and promotes telomere replication in a wide range of eukaryotes [12–16]. Budding yeast CST (Cdc13-Stn1-Ten1) binds the G-overhang where it protects the telomere, recruits telomerase and mediates C-strand fill-in [17–20]. Mammalian CST (CTC1-STN1-TEN1) is less important for telomere-end protection but it functions both in telomere duplex replication and C-strand fill-in [21–25]. It is also proposed to limit telomerase action, perhaps by competing for binding to the telomere protein TPP1 [26,27].

CST has additional genome-wide roles that are just starting to be appreciated [13,24,28–31]. In humans, CST facilitates recovery from various forms of replication stress throughout the genome. It promotes activation of dormant or late firing origins in response to replication fork stalling [24] and enhances viability when cells are treated with drugs that block replication fork progression [30]. Mutations in CTC1 cause the diseases Coats plus and dyskeratosis congenita [32–34]. The telomeric and non-telomeric roles of CST are likely to underlie the severity of these diseases.

Although the mechanism of CST action is still unclear, multiple studies indicate a link to pol α. Mammalian CTC1 and STN1 were originally identified as Alpha Accessory Factor (AAF), a factor that co-purified with pol α and enhanced its processivity and affinity for ssDNA templates [35,36]. CST and pol α have since been shown to interact in yeast, plants and mammals [20,21,37,38]. *Xenopus* CST stimulates DNA priming by pol α on ssDNA [39] while *Candida* CST enhances primase activity and primase to polymerase switching [40].

**Fig 1:**
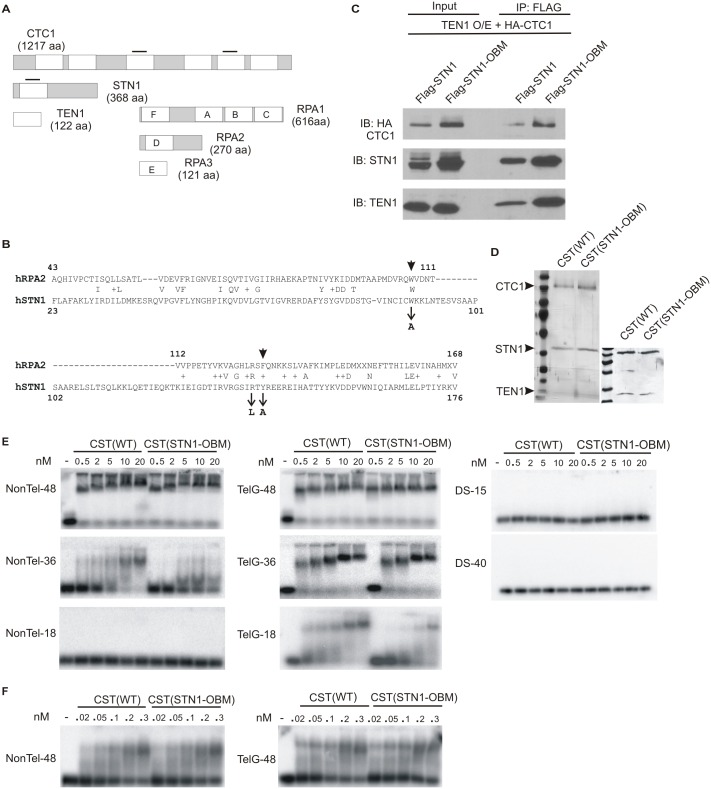
STN1 OB fold mutation affects DNA binding but not CST complex formation. (A) Cartoon depicting known and predicted OB fold domains in RPA and CST, white boxes indicate OB folds. Black bars mark OB folds in CST (known for STN1, predicted for CTC1, see Materials and Methods) where mutations alter DNA binding [15,36]. (B) Alignment of human STN1 and RPA32 OB folds showing identical and similar (+) residues [52]. Arrows indicate mutations in the STN1 OB fold mutant (STN1-OBM), arrowheads mark corresponding RPA residues that contact DNA directly in the *U*. *maydis* crystal structure [41]. (C) Western blots showing co-immunoprecipitation of CTC1 and TEN1 with STN1 or STN1-OBM using extracts from TEN1 overexpressing HeLa cells transiently transfected with HA-CTC1 and FLAG-STN1 or FLAG-STN1-OBM. (D) Silver-stained gels showing co-purification of FLAG-CTC1 and TEN1 with His-STN1 or His-STN1-OBM expressed in insect cells. Left, a full 12% gel showing all three subunits. Right, portion of a more heavily stained 15% gel showing STN1 and TEN1. (E-F) EMSAs showing CST(WT) or CST(STN1-OBM) binding to non-telomeric (NonTel) or telomeric G-strand (TelG) oligonucleotides or dsDNA (DS) of the indicated lengths. Increasing concentrations of CST were incubated with 0.1 nM labeled DNA for 30 min prior to separation in agarose gels. (E) The same CST(WT) or CST(STN1-OBM) preparation was used in all the EMSAs with ssDNA.

CST exhibits notable structural similarities to Replication Protein A (RPA) the eukaryotic ssDNA binding protein that directs the assembly of multi-protein complexes needed for DNA replication, recombination and repair (Fig 1A) [23]. RPA has three subunits (RPA1, RPA2 and RPA3) that together harbor six OB (oligonucleotide-oligosaccharide binding) folds (Fig 1A) [41,42]. Four of the OB folds participate in DNA binding. Because RPA has multiple DNA binding sites, individual OB folds can undergo rapid dissociation and re-association without causing the protein to fall off the DNA [43,44]. This dissociation and re-association of OB folds underlies RPA function as it makes binding dynamic and enables RPA to diffuse along DNA to melt DNA structure or load and unload proteins needed for replication, recombination or repair [45].

Like RPA, CST appears to harbor OB folds in all three subunits and X-ray crystallography indicates striking structural similarity between STN1-TEN1 and RPA2-RPA3 dimers [46–48]. The structural conservation encompasses the OB-fold and winged helix domains and the dimerization interface. The large subunits of RPA and CST appear less well conserved. Although RPA1 and Cdc13 from budding yeast each harbor 4 OB folds, Cdc13 needs only one OB fold for high affinity binding [42,49]. Moreover, Cdc13 dimerizes through its N-terminal OB fold to form a DNA pol α binding surface, whereas RPA1 does not self-associate [50]. In mammalian cells, Cdc13 is replaced by CTC1 but the two proteins share little sequence identity and the extent of structural or functional conservation is unclear [37]. Protein threading programs (Phyr2 and HHpred) predict 5–6 OB folds in human CTC1 with the three most C-terminal folds resembling those of RPA1 (Fig 1A). *In vitro* studies have revealed an additional parallel between RPA and human CST as in each case high affinity DNA binding requires formation of the three protein complex [23,26,41,42]. The structural similarities between RPA and CST raise the possibility that dynamic DNA binding through multiple OB folds may also contribute to CST function.

Since so little is known about CST mechanism of action and the relationship between DNA binding and CST function, we set out to analyze how reduced DNA binding affects CST activity at telomeres and elsewhere in the genome. We describe a STN1 OB fold mutant that preferentially affects *in vitro* binding to short DNA substrates. *In vivo*, the mutant can substitute for wild type STN1 in some aspects of CST function but other aspects are impaired. DNA binding studies indicate that, like RPA, CST appears to contact DNA via multiple OB folds and to have distinct modes of binding. However, we also provide evidence that the organization of OB-fold engagement by CST is quite different.

## Results

### Alteration of DNA-binding by STN1 OB-fold mutation

To generate the STN1 OB-fold mutant (STN1-OBM) we changed three residues (W89A, R139L, Y141A) that are conserved between STN1 and the OB fold of RPA2 and which either directly contact, or lie very close to DNA in RPA crystal structures (Fig 1B) [41,51]. The W89A and Y141A mutations were chosen because the equivalent mutations in mouse STN1/AAF-44 reduced DNA binding by ~60% in pull-down assays with biotin-labeled poly-dC [36]

Co-immunoprecipitation and tandem affinity purification experiments verified that the STN1 mutant retained the ability to form a complex with CTC1 and TEN1 (Fig 1C and 1D). In initial experiments, we co-expressed FLAG-tagged STN1 or STN1-OBM with HA-CTC1 in a previously characterized HeLa cell line over-expressing TEN1 [30]. When STN1-OBM was immunoprecipitated from whole cell lysate, both CTC1 and TEN1 co-purified (Fig 1C). We also generated recombinant CST complexes containing wild type STN1 (CST(WT)) or STN1-OBM (CST(STN1-OBM)) by co-infecting insect cells with baculovirus encoding FLAG-tagged CTC1, untagged TEN1 and His-tagged STN1 or STN1-OBM. Protein complexes were affinity purified on nickel resin followed by FLAG beads (Fig 1D) and again CTC1 and TEN1 co-purified with STN1-OBM.

We next examined how STN1-OBM affects the ability of CST to bind a range of DNA substrates. As the affinity of CST for short versus long substrates seems to depend on DNA sequence [26], we monitored binding of CST(WT) and CST(STN1-OBM) to telomeric and non-telomeric oligonucleotides of various lengths (Fig 1E and 1F and Table 1). When we used electrophoretic mobility shift assays (EMSA) to compare binding of CST(WT) and CST(STN1-OBM) to 48 nt substrates, the two complexes appeared to bind both non-telomeric (NonTel-48) and telomeric G-strand (TelG-48) DNA with similar affinity. However relative to CST(WT), the CST(STN1-OBM)) bound less efficiently to the 36 nt non-telomeric (NonTel-36) and the 18 nt telomeric G-strand (TelG-18) substrates. Neither complex bound equivalent concentrations of the 18 nt non-telomeric oligonucleotide (NonTel-18) or dsDNA (Fig 1E) [23]. These results suggest that the STN1-OBM preferentially affects binding to short substrates. Note, we refer to TelG-18 as a short substrate because CST has very low affinity for DNA with fewer telomeric repeats, e.g. TelG-12 [26]. Our results also confirm that CST binds both telomeric and non-telomeric DNA but that telomeric DNA is preferred when substrate length is short [26].

**Table 1:** Sequence of oligonucleotides used in DNA binding assays.

Oligo	Sequence (5’-3’)
TelG-18	GGTTAGGGTTAGGGTTAG
TelG-36	GGTTAGGGTTAGGGTTAGGGTTAGGGTTAGGGTTAG
TelG-48	GGTTAGGGTTAGGGTTAGGGTTAGGGTTAGGGTTAGGGTTAGGGTTAG
NonTel-18	AGCGTATCCGTTCAGTTG
NonTel-36	AGCGTATCCGTTCAGTTGAGCGTATCCGTTCAGTTG
NonTel-48	AGCGTATCCGTTCAGTTGAGCGTATCCGTTCAGTTGAGCGTATCCGTT
ds 15	TTCGATCTACGTCAGCA 5’ T ............... TTGCTAGATGCAGTCGT 3’
ds 40	TTTACGTCAGCACGATCTACGTCAGCACGATCTACGTCAGCA 5’ T ........................................ TTATGCAGTCGTGCTAGATGCAGTCGTGCTAGATGCAGTCGT 3’

### STN1-OBM fails to rescue to anaphase bridges after endogenous STN1 depletion

**Fig 2:**
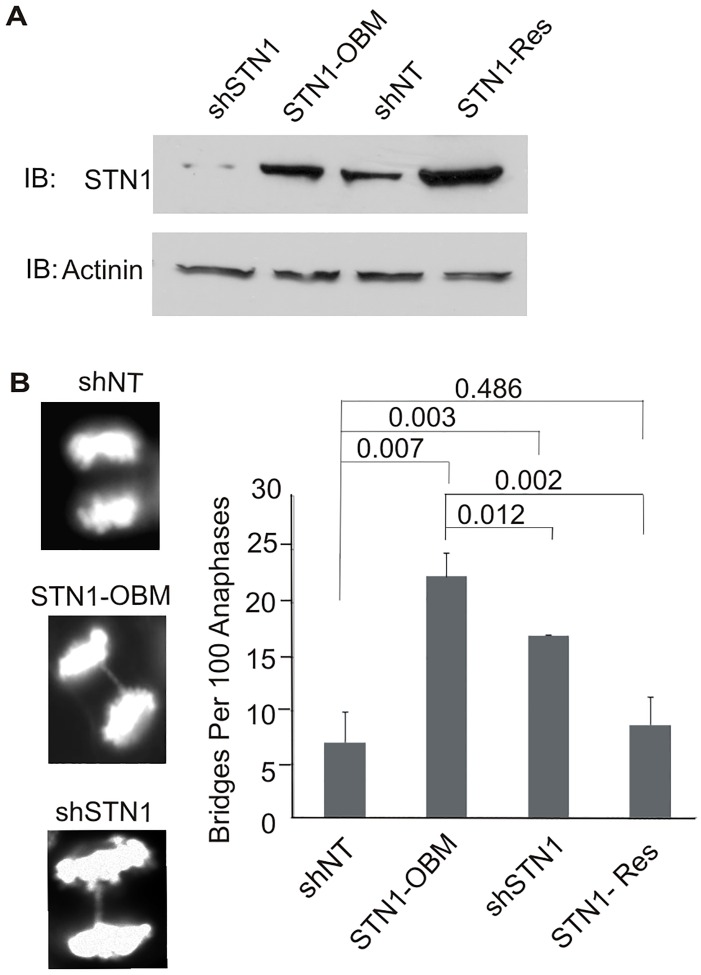
*In vivo* expression of STN1-OBM causes anaphase bridges. (A) Western blot showing levels of STN1 in HeLa cells expressing non-target shRNA (shNT) or STN1 shRNA (shSTN1) and shSTN1 cells with sh-resistant mutant STN1 (STN1-OBM) or wild type STN1 (STN1-Res). Blot was probed with antibody to STN1 or to actinin for a loading control. (B) Left; representative images of DAPI-stained anaphase cells with/ without bridges. Anaphase cells with no bridge in shNT (top) and with bridges in shSTN1 and STN1-OBM cells (middle and bottom). Right; quantification of bridges (200 anaphases counted per cell line per experiment. n = 3 experiments, mean ± S.E.M, p-values are indicated above bars).

To examine the *in vivo* effects of STN1 OB fold mutation, we generated HeLa cells that stably express FLAG-tagged STN1-OBM (Fig 2A and S1A Fig) by introducing an shRNA-resistant STN1-OBM cDNA into a previously characterized HeLa cell line expressing STN1 shRNA (shSTN1) [24,25]. A cell line expressing FLAG-tagged shRNA-resistant wild type STN1 (STN1-Res) was previously made in the same manner [24].

In initial experiments, we asked if STN1-OBM could rescue the increase in anaphase bridges that occurs after STN1 depletion [24]. STN1-OBM cells and a series of control cells (shSTN1, STN1-Res and shNT, a non-target shRNA control) were arrested in mitosis with nocadozole, released for 45–60 min, fixed and scored for the number of anaphase cells with DAPI-stained bridges (Fig 2B). As previously described, depletion of STN1 caused an increase in bridges and this was rescued by expression of sh-resistant wild type STN1 [24]. In contrast, expression of sh-resistant STN1-OBM did not rescue bridge formation but instead further increased the fraction of anaphase cells with bridges. The reason for the higher level of bridges in the STN1-OBM cells relative to the shSTN1 cells is unclear but a possible cause is that STN1-OBM replaces residual endogenous STN1 in CST complexes. Overall, the inability of STN1-OBM to rescue the anaphase bridge phenotype indicates that STN1-OBM cannot substitute for wild type STN1 in some aspects of CST function.

### STN1-OBM affects telomere duplex replication but not C-strand fill-in

**Fig 3:**
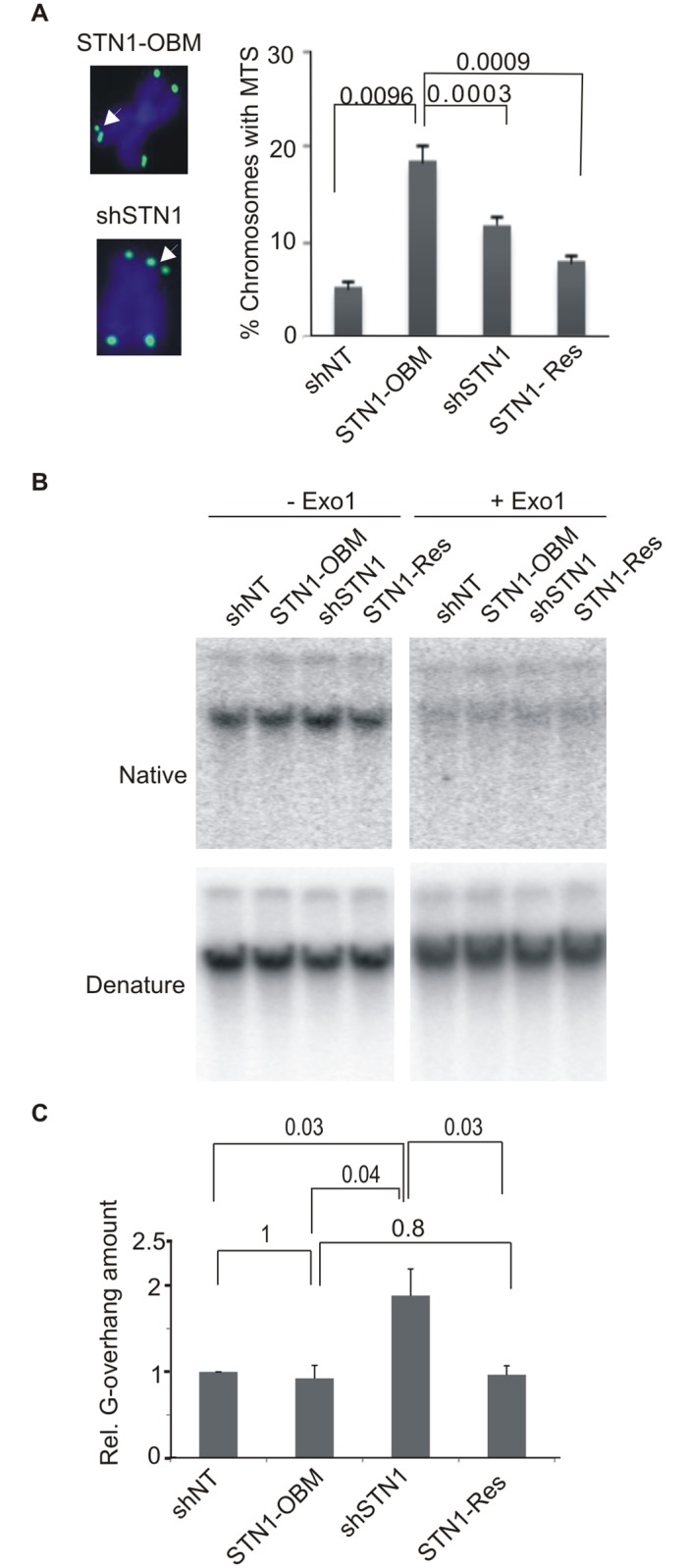
STN1-OBM causes multiple telomere signals (MTS) but does not affect G-overhang maintenance. (A) Telomere FISH of STN1-OBM or shSTN1 cells. Left; representative images of single metaphase chromosomes. White arrows, MTS; green, FITC-(C_3_TA_2_)_3_ probe; blue, DAPI. Right; Quantification of MTS (n = 4 experiments mean ± S.E.M.). Individual chromosomes were scored positive for MTS if they had MTS at one or more telomeres. (B-C) G-overhang abundance in asynchronous cells monitored by in-gel hybridization with (A_2_TC_3_)_4_ probe. (B) Representative gels showing hybridization to genomic DNA from the indicated cells under native or denaturing conditions. (C) Quantification of G-overhang abundance in asynchronous cells (n = 3 experiments, mean ± S.E.M., p-values are indicated above bars).

Anaphase bridges can have a number of causes including telomere-to-telomere fusion and the presence of unresolved replication intermediates either at telomeres or elsewhere in the genome [53,54]. Thus, to ask more specifically whether STN1-OBM affects the telomeric roles of CST, we looked for changes in telomere structure. Metaphase spreads from STN1-OBM and control cells were hybridized with telomere probe and examined for telomere loss, telomere fusions or other abnormal telomere signals. As previously reported, we did not observe an increase in telomere loss or telomere fusions in the shSTN1 and STN1-Res cells [24] (S1B Fig). This was also true for the STN1-OBM cells (S1B Fig), indicating that the anaphase bridges caused by STN1-OBM expression are unlikely to be caused by telomere fusions. However, relative to the STN1-Res control, the STN1-OBM cells showed a large increase in individual chromatids exhibiting Multiple Telomeric FISH Signals (MTS) (Fig 3A). As expected, the shSTN1 cells also showed an increase in MTS but it was lower than in the STN1-OBM cells. Again this may reflect the displacement of residual endogenous STN1 with STN1-OBM in CST complexes. Past studies have shown that MTS arise after fork stalling during replication of the telomere duplex [8] and they occur after depletion of the various factors needed for telomere replication, including CST [8,25]. In particular, STN1 depletion slows replication through the telomere duplex and causes the appearance of MTS [25]. We therefore conclude that STN1-OBM is unable to rescue the deficiency in telomere duplex replication caused by STN1 depletion.

We next asked if STN1-OBM affects telomere length or G-overhang structure. Genomic DNA was isolated from STN1-OBM or control cells and telomere restriction fragments were examined by Southern blotting or in-gel hybridization to monitor telomere length (S1C Fig). This analysis revealed that telomeres of shSTN1, STN1-Res and STN1-OBM cells were very similar in length, thus confirming our previous finding that telomere length in HeLa cells is largely unaffected by STN1 knockdown [25] and indicating that STN1-OBM has dominant negative effect. We then used an in-gel hybridization assay to ask if STN1-OBM affects G-overhang structure. Restriction digested DNA was separated briefly in agarose gels and hybridized with a probe to the telomeric G-strand under non-denaturing conditions (Fig 3B). The DNA was then denatured and re-hybridized with the same probe. Quantification of the overhang signal relative to total telomeric DNA revealed the expected increase in overhang amount in the STN1-depleted cells (Fig 3C). This increase has previously been shown to result from inefficient C-strand fill-in following telomerase extension [21,25]. Given that STN1-OBM affects binding to telomeric G-strand DNA *in vitro* (Fig 1E) and telomere duplex replication *in vivo* (Fig 2), we anticipated that the STN1-OBM cells would also have a deficiency in C-strand fill-in. However, to our surprise we found that STN1-OBM cells had normal length overhangs (Fig 3C) implying that the mutant STN1 was able to rescue C-strand fill-in.

**Fig 4:**
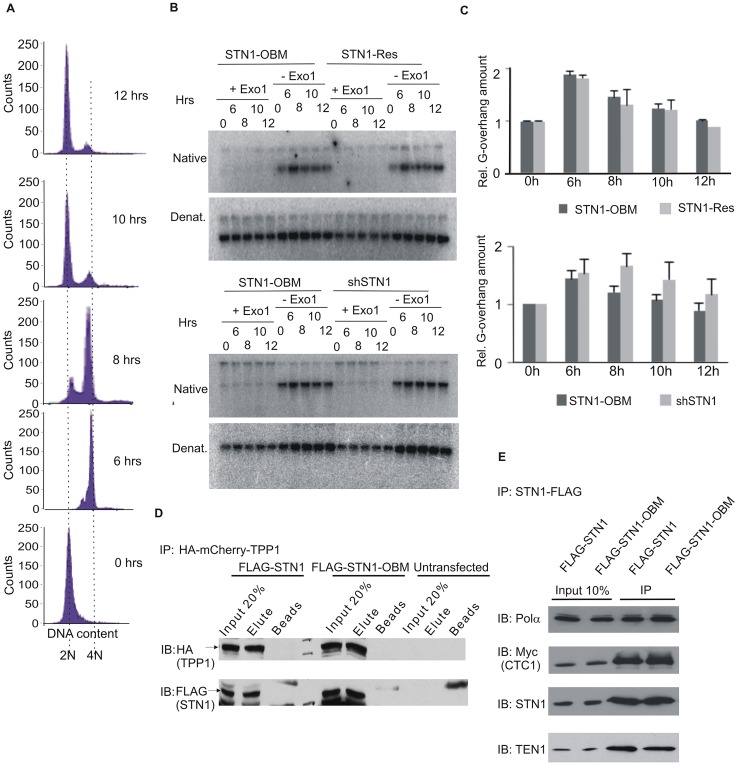
STN1-OBM is competent for C-strand fill-in and TPP1 and pol α interaction. (A-C) Analysis of C-strand fill-in. (A) FACS analysis of DNA content showing synchrony of STN1-OBM cells used in (B). (B) Representative gels showing in-gel hybridization of (TA_2_C_3_)_4_ probe to DNA from cells harvested at the indicated times after release from G1/S block. (C) Quantification of G-overhang abundance. Cell types were analyzed in pairs, n = 3 experiments for shSTN1 + STN1-OBM, mean ± S.E.M.; n = 2 experiments for STN1-OBM + STN1-Res, error bars show min/max values). (D) Western blot showing co-immunoprecipitation of TPP1 with STN1 or STN1-OBM. Cells were transfected with FLAG-STN1 or FLAG-STN1-OBM plus HA-mCherry-TPP1 expression constructs. TPP1 was precipitated with antibody to HA. (E) Co-immunoprecipitation of DNA pol α with CST. Cells were transfected with FLAG-STN1 or FLAG-STN1-OBM, Myc-CTC1 and TEN1. CST was precipitated with FLAG beads.

G-overhang length is determined by a number of activities that occur at specific stages in the cell cycle. Overhangs are elongated in S-phase as a result of G-strand synthesis by telomerase and C-strand resection by nuclease [6,7]. They are then returned to their original length in late S/G2 via C-strand fill-in by DNA pol α [25]. Given this balance between activities, it was possible that the normal length overhangs in the STN1-OBM cells result from decreased G-strand extension in S-phase in combination with decreased C-strand fill-in during late S/G2. To investigate this possibility, we examined G-overhang length dynamics during the cell cycle. Cells were synchronized in G1/S with a double thymidine block, released into S-phase and harvested at intervals as they passed through mid S-phase, G2/M and back into G1 (Fig 4A and S2A Fig). Following DNA isolation, relative overhang length was examined by in-gel hybridization as described above (Fig 4B and 4C). Quantification of the overhang signal indicated that the STN1-Res cells showed the expected increase in overhang abundance as they transitioned from G1 (0 hr) into mid S-phase (6 hrs) [6,7,25]. The overhang signal then declined due to C-strand fill-in as the cells transitioned into G2 (8 hrs) and G1 of the next cell cycle (10–12 hrs) [25,55]. Interestingly, the pattern of overhang elongation and shortening in the STN1-OBM cells was indistinguishable from that seen with the control STN1-Res cells indicating that STN1-OBM does not affect overhang elongation or C-strand fill-in. In contrast, the shSTN1 cells exhibited the expected delay in overhang shortening in late S/G2 reflecting the deficiency in C-strand fill-in [25]. Thus although STN1-OBM affects telomere duplex replication, it does not appear to affect C-strand fill-in by DNA pol α.

### STN1-OBM does not disrupt interaction with TPP1 or DNA pol α

Several studies have shown that STN1 can interact with the shelterin protein TPP1 [26,56], suggesting that this interaction might be important for recruiting CST or stabilizing CST binding at the telomere. Given that OB folds can mediate protein-protein interactions as well as DNA binding [42], we considered the possibility that the *in vivo* effects of STN1-OBM expression might reflect decreased binding of CST to TPP1. To test for a disruption in the TPP1-STN1 interaction, we transfected 293T cells with constructs encoding FLAG-tagged STN1 or STN1-OBM and HA-mCherry tagged TPP1 [57] and monitored co-immunoprecipitation of TPP1 with STN1. When TPP1 was precipitated with antibody to HA, Western blot analysis showed that the STN1 and STN1-OBM co-precipitated with equivalent efficiency (Fig 4D). We therefore conclude that STN1-OBM retains the ability to bind TPP1. We also tested whether STN1-OBM disrupts binding to DNA pol α, the only other known CST binding partner [21,58]. 293T cells were transfected with constructs encoding TEN1, FLAG or Myc-tagged CTC1 and FLAG-STN1 or FLAG-STN1-OBM, and CST was then precipitated from extracts with FLAG antibody. Western blot analysis of the immunoprecipitates showed that pol α co-precipitated with FLAG-STN1 only if both CTC1 and STN1 were overexpressed (S2B Fig). However the level of pol α precipitation was similar with CST(WT) and CST(STN1-OBM), indicating that STN1-OBM does not prevent CST from binding to pol α (Fig 4E and S2B Fig). Our finding that C-strand fill-in is unaffected by STN1-OBM (Figs 3B and 4C) provides further support for a functional interaction between pol α and CST(STN1-OBM),

### STN1-OBM can function in replication rescue after genome wide replication fork stalling

**Fig 5:**
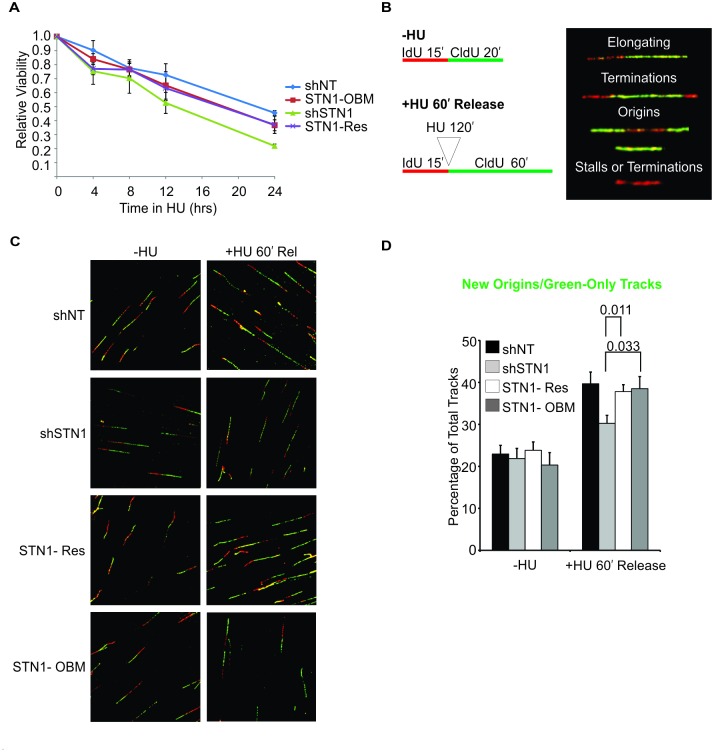
STN1-OBM rescues viability and restores origin firing after HU treatment. (A) MTT assay showing viability after HU treatment. Cells were treated with 2 mM HU for the indicated times and harvested for MTT assay 24 hrs later. Values are relative to untreated cells of the same cell type. Each time point was assayed in triplicate and the data are shown as the mean ± S.D from 3 independent experiments. For each cell line, the value of the untreated sample was set at 1. (B-D) DNA fiber analysis of origin firing following release from 2 mM HU. (B) Left: schematic showing timing of IdU and CldU labeling relative to HU treatment. Right: types of replication event scored. (C) Representative images of DNA tracks. Red, IdU; Green, CldU. (D) Graph indicating the percent of DNA tracks showing new origin firing (green-only tracks) (n = 7 experiments, mean ± S.E.M, p-values are indicated above bars).

Since the above studies indicate that STN1-OBM has selective effects on CST function, we next examined whether the mutant affects the response to genome-wide replication fork stalling. In initial experiments, we asked if STN1-OBM could substitute for wild type STN1 to maintain cell viability after HU (hydroxyurea) treatment. STN1-OBM and control cells were treated with 2 mM HU for 0–24 hrs, allowed to recover for 24 hrs then cell viability was monitored by MTT assay (Fig 5A). As observed previously, STN1 depletion increased sensitivity to HU [30]. However, wild type STN1 (STN1-Res) and STN1-OBM rescued this sensitivity to an equal extent, indicating the mutant was sufficient to allow CST function in recovery from prolonged fork stalling.

To further explore the effect of STN1-OBM on recovery from fork stalling, we performed DNA fiber analysis to determine if the mutant can substitute for endogenous STN1 to promote origin firing after HU treatment. Cells were labeled with IdU (iododeoxyuridine) for 15 minutes, treated with HU for two hours then released into media containing CldU (chlorodeoxyuridine) for 60 min (Fig 5B). The cells were then collected, lysed and the DNA fibers spread on silanized slides by hydrodynamic flow [59]. The fibers were stained with antibody to IdU and CldU then visualized by confocal microscopy to score the replication events (Fig 5B–5D and S3 Fig). As observed previously, the HU-treated shSTN1 cells exhibited fewer green-only (CldU-only) tracks than the shNT and STN1-Res control cells [24,30], indicating that STN1 depletion caused a decrease in new origin firing after HU release. In contrast, the HU-treated STN1-OBM cells exhibited a similar number of green-only tracks to the control cells. The frequency of other replication events was also similar (S3 Fig). We therefore conclude that the STN1-OBM can substitute for wild type STN1 to promote new origin firing. Overall, our results indicate that STN1-OBM does not affect the capacity of CST to aid in the restart of replication following exogenous replication stress. This is in direct contrast to the inability of STN1-OBM to rescue the effects of endogenous stress as seen by the increase in anaphase bridges and MTS in unchallenged STN1-OBM cells.

### Effects of STN1-OBM on binding affinity and stability

Our finding that STN-OBM affects only specific aspects of CST function is analogous to what has been observed for certain RPA OB-fold mutants, which support DNA replication but are defective for DNA repair [60,61]. These mutants cause only a small decrease in overall affinity of RPA for ssDNA and the deficit in repair is thought to result from a change in the dynamics of RPA binding through its multiple OB folds [45,61]. The structural similarities between CST and RPA suggest that CST function could also rely on dynamic binding using multiple OB folds. We therefore set out to explore the extent to which RPA binding can be used as a paradigm for understanding CST activity and the *in vivo* separation of function observed with STN1-OBM.

**Fig 6:**
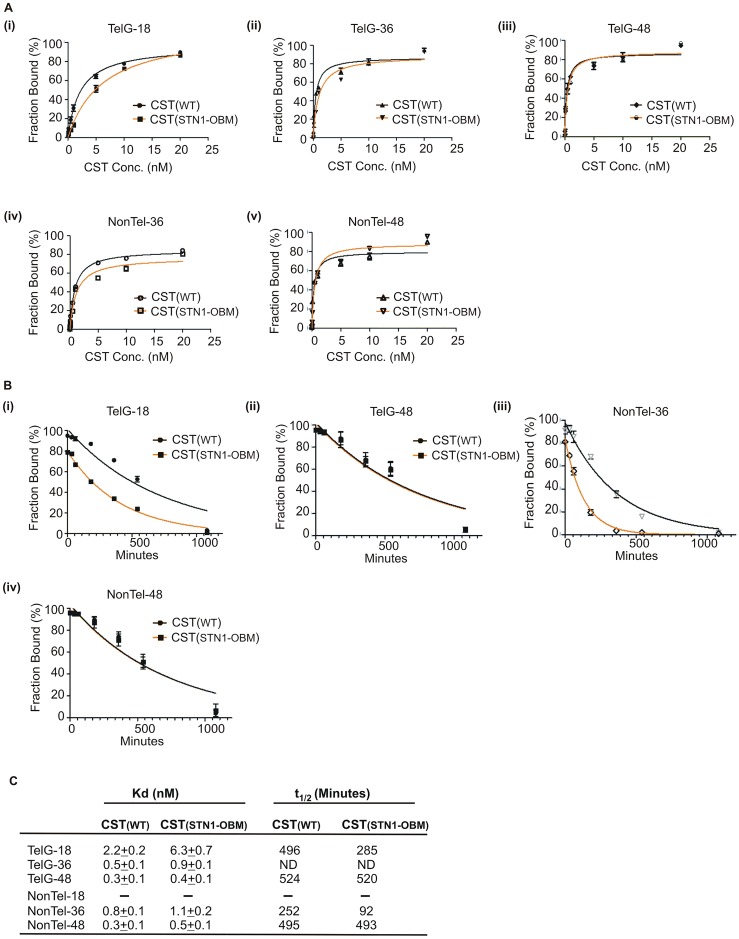
Analysis of CST DNA binding parameters. (A) Binding isotherms used to determine apparent dissociation constants for CST(WT) or CST(STN1-OBM) and the indicated ssDNA substrates. Data were obtained by filter binding assay and fit to a one site specific binding model. Mean ± SEM, n = 3 independent experiments each with a different protein preparation. (B) Dissociation kinetics for CST bound to the indicated substrates The fraction of labeled DNA remaining bound was determined by filter binding at the indicated times. Data were fit to a one phase exponential decay equation to obtain the dissociation rate (t½)). Mean ± SEM, n = 3 independent experiments. (C) Table summarizing Kd(app) and t½ for CST(WT) or CST(STN1-OBM) and the indicated ssDNA substrates.—undetectable binding, ND: not determined.

As a first step, we revisited the effect of STN1-OBM on DNA binding by using filter binding assays to quantify the affinity of CST(WT) and CST(STN1-OBM) for telomeric and non-telomeric substrates of various lengths (Fig 6A, 6C and S4 Fig). CST purified from insect cells was incubated with ^32^P-labeled DNA then the DNA-protein complexes were separated from free DNA by filtration through a sandwich of nitrocellulose and HyBond membrane. The bound versus free DNA was quantified and used to calculate the apparent dissociation constant (Kd,app). Despite the different approach used to separate bound from free DNA in the filter binding and the original gel shift assay (Fig 1E), the results of the two assays were qualitatively similar. The filter binding indicated that CST(WT) and CST(STN1-OBM) bound the 48 nt telomeric and non-telomeric substrates with a similar Kd,app while binding to TelG-18 was decreased for CST(STN1-OBM) relative to CST(WT) (Fig 6A and 6C). Thus, the filter binding analysis again indicated that STN1-OBM preferentially affects binding to short DNA substrates. However the analysis also revealed that the overall decrease in Kd,app for CST(STN1-OBM) binding to TelG-18 was only 2–3 fold.

When gel shift assays were used to examine CST(STN1-OBM) binding to the TelG-18 and NonTel-36 substrates a substantial amount of DNA was seen to migrate between the bands corresponding to free DNA and CST-bound DNA (Fig 1E). This observation suggested that the DNA-protein complexes were dissociating and hence the decrease in Kd,app for CST(STN1-OBM) might reflect less stable binding. To test this possibility, we measured the rate of CST(WT) and CST(STN1-OBM) dissociation (t½) from selected substrates. CST complexes were bound to ^32^P-labeled oligonucleotide, challenged with an excess of the corresponding cold oligonucleotide for various times and the remaining labeled DNA/protein complex was quantified by filter binding. This experiment revealed that CST(STN1-OBM) dissociated from the labeled TelG-18 and NonTel-36 1.6–2.6-fold faster than CST(WT) whereas dissociation from TelG-48 and NonTel-48 was essentially the same (Fig 6B and 6C). We therefore conclude that the STN1-OB fold acts to stabilize CST binding to short ssDNA substrates.

The 2–3 fold decrease in affinity of CST(STN1-OBM) for TelG-18 resembles the modest decrease in RPA affinity for ssDNA that has been observed after mutation of individual OB folds [60,62]. In the case of RPA, the small effect on overall binding affinity reflects the presence of multiple DNA binding domains within the complex such that disruption of one binding domain has a small effect on the macroscopic affinity constant. Thus, the observed decrease in CST(STN1-OBM) binding fits with the model that CST also engages DNA via multiple DNA binding domains. Given the six predicted OB folds in CTC1, we anticipate that these multiple DNA binding domains correspond to the STN1 OB fold plus some or all of the OB folds in CTC1.

### CST subunit interactions with ssDNA substrates

While CST appears to resemble RPA in terms of subunit composition and utilization of multiple OB folds for DNA binding, our finding that CST(STN1-OBM) destabilizes binding to short oligonucleotides suggested a significant difference in how the two complexes bind short DNA substrates (Fig 6C). RPA binds DNA in a 5’ to 3’ direction with the OB-folds of RPA1 contacting DNA towards the 5’ end and providing the highest affinity binding sites [41,62]. As a result, OB-A and OB-B of RPA1 provide the only contacts to an 8 nt substrate. OB-A, -B and–C of RPA1 contact substrates of 12–23 nt but RPA2 (the STN1 equivalent) only contacts longer substrates of ~30 nt [41,42]. Consequently, mutations in RPA2 OB-D affect binding to long rather than short ssDNA [60,62]. Our finding that STN1-OBM destabilizes binding to short (e.g. TelG-18) but not long (TelG-48 & NonTel-48) substrates (Fig 6C) suggested that, unlike RPA2, STN1 directly engages the DNA of short substrates to stabilize binding.

**Fig 7:**
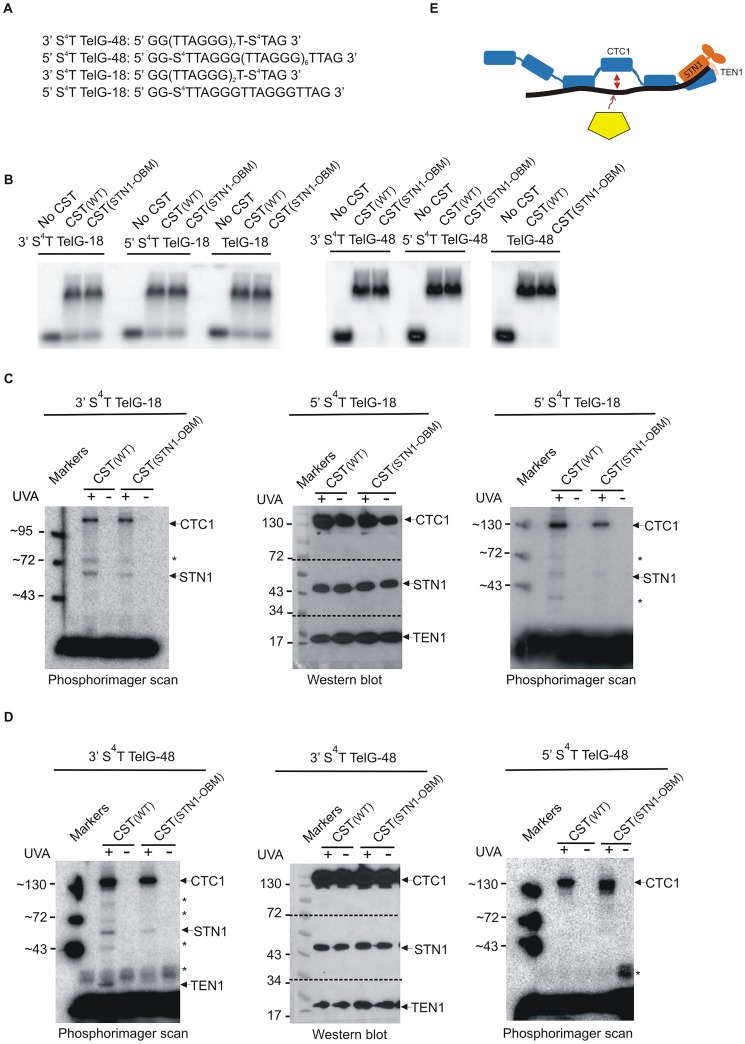
Photo-crosslinking of CST subunits to thiothymidine substituted ssDNA. (A) Positions of s^4^T substitutions in TelG-18 and TelG-48 DNA substrates. (B) EMSA showing the s^4^T substitutions do not affect CST(WT) or CST(STN1-OBM) binding to TelG-18 or TelG-48. (C-D) Products obtained after photo-crosslinking. Left and right panels: Phosphorimager scans showing ^32^P-labeled cross-linking products. Central panels: Western blots from the same gels showing positions of uncross-linked CTC1, STN1 and TEN1. (C) Products obtained with 3’ (left) or 5’ (right) modified TelG-18. (D) Products obtained with 3’ (left) or 5’ (right) modified TelG-48. * indicates cross-linking products observed only in some experiments. They may represent CTC1 or STN1 degradation products. Markers on the phosphorimager scans were obtained by laying the gels on nitrocellulose membrane and marking the positions of the marker bands with radioactive ink. For the Western blots, the membrane was cut into pieces, probed with antibody to CTC1, STN1 or TEN1, reassembled and exposed to film. The film was laid over the membrane and photographed to visualize both the markers and the CST bands. (E) Dynamic binding model of CST showing micro-dissociation of an individual OB fold (blue) to allow binding of an alternative protein (yellow).

To further explore this possibility, we used photo-crosslinking to explore the proximity of individual CST subunits to the 5’ or 3’ ends of 18 or 48 nt TelG oligonucleotides. CST(WT) and CST(STN1-OBM) were incubated with ^32^P-labeled TelG-18 or TelG-48 that had a photoactivatable 4-thiothymidine (s^4^T) at the third nucleotide from the 5’ or 3’ end (Fig 7A and S5 Fig). The DNA-protein complexes were cross-linked by irradiation with UVA and then separated in a SDS-polyacrylamide gel. The gel was scanned by phosphorimager to determine whether CTC1, STN1 or TEN1 had been cross-linked to the labeled DNA. Equivalent UV-irradiated samples separated in the same gel were used for Western blot analysis to determine the positions of uncross-linked CTC1, STN1 and TEN1. The low level of cross-linking precluded detection of the cross-linked DNA-protein complexes by Western blot. Additional reactions that had not been subject to cross-linking were analyzed by EMSA to monitor DNA binding. As shown in Fig 7B, the s^4^T residues did not significantly alter CST(WT) or CST(STN1-OBM) binding to either substrate.

Analysis of the crosslinking products obtained with CST(WT) and 5’- or 3’-s^4^T TelG-18 revealed labeled bands that migrated at positions expected for CTC1 (≥130 kD) and STN1 (>43 kD) (Fig 7C) indicating cross-linking to either substrate. However, cross-linking of STN1 relative to CTC1 was less efficient with the 5’-s^4^T TelG-18, suggesting that STN1 was positioned closer to the DNA 3’ end. It was not possible to tell if TEN1 was cross-linked to either substrate because TEN1 migrated in the same region of the gel as the uncross-linked DNA. Thus, bands corresponding to TEN1-TelG-18 may be obscured by the heavy signal from the uncross-linked DNA. Overall, the results indicate that binding of CST to a short 18 nt substrate positions the DNA in close proximity to STN1. Comparison of the cross-linking products obtained with the 3’ modified TelG-18 and CST(WT) or CST(STN1-OBM) revealed that cross-linking to STN1-OBM was reduced relative to wild type STN1. This finding indicates that the contacts between STN1 and DNA are altered by STN1-OBM.

Analysis of the products obtained with CST(WT) bound to TelG-48 revealed that only CTC1 was reproducibly cross-linked to the 5’-s^4^T substrate. In contrast, the 3’-s^4^T substrate crosslinked to all three CST subunits. CTC1 cross-linked more efficiently than STN1 or TEN1 and the level of TEN1 cross-linking was somewhat variable (Fig 7D) (note: cross-linking of TEN1 to TelG-48 retards TEN1 migration enough for the band from the cross-linked product to become visible above the uncross-linked DNA). The cross-linking of STN1 and TEN1 to the 3’-s^4^T substrate but not the 5’-s^4^T substrate indicates that both subunits must be in close proximity to the DNA 3’ terminus but not the 5’ terminus. Examination of the photo-products obtained with of CST(STN1-OBM) bound to 3’-s^4^T TelG-48 revealed less cross-linking to STN1 and TEN1 relative to CST(WT) but CTC1 photoproducts were still formed, again indicating that STN1-OBM alters how STN1 contacts DNA.

Taken together the above results demonstrate that CST(WT) binds long substrates with the DNA 3’ end positioned close to the CTC1-STN1-TEN1 interface while the 5’ end only contacts CTC1. We therefore infer that, CST binds DNA in a similar orientation to RPA: i.e. with the large subunits of each complex contacting DNA near the 5’ end and the two smaller subunits positioned at the 3’ end. However, our data indicate that the identity of the binding sites used to engage short DNA substrates differs between CST and RPA. For CST, the binding sites lie close to the interface between CTC1, STN1 and TEN1, they engage DNA toward the 3’ end, and STN1 plays an important role in stabilizing the interaction. For RPA, the primary binding sites for short substrates are OB-A and OB-B of RPA1 and these engage DNA at the 5’ end. Thus, despite sharing some common structural features CST and RPA engage DNA quite differently.

In addition to addressing the architecture of CST-DNA complexes, the *in vitro* cross-linking studies combine with the analysis of DNA binding affinity start to explain the *in vivo* separation of function observed with STN1-OBM cells. Our results show that the STN1 OB-fold mutation alters the interaction between STN1 and ssDNA and this translates into altered binding of CST to some but not all DNA substrates. *In vivo*, CST is likely to encounter DNA substrates of varied length and structure as the complex helps resolve a wide range of replication problems at telomeres and genome-wide. Thus, similar to what has been observed for RPA OB-fold mutants [60,61], the altered DNA binding caused by STN1-OBM is likely to impair the ability of CST to bind and mediate the resolution of only a subset of replication intermediates.

## Discussion

Here we describe a series of *in vivo* and *in vitro* experiments that address the mechanism of CST action at telomeres and elsewhere in the genome. We show that a STN1-OB-fold mutant (STN1-OBM) which preferentially decreases affinity of CST for short ssDNA substrates is competent for some aspects of CST function but deficient in others. The effects of STN1-OBM do not align with the telomeric versus non-telomeric roles of CST, but instead separate out the different aspects of CST function both during telomere replication and in genome-wide replication rescue. At telomeres, STN1-OBM cells are competent for C-strand fill-in following telomerase action but they exhibit increased MTS which are indicative of deficiencies in the earlier process of telomere duplex replication. STN1-OBM cells are also competent to restart replication via new origin firing following exogenous genome-wide replication stress. However, STN1-OBM is not able to prevent the accumulation of anaphase bridges during mitosis. The latter finding indicates a deficiency in genome-wide resolution of endogenous replication stress because the anaphase bridges caused by CST depletion occur at both telomeric and non-telomeric loci [27,30]. Our findings underscore the importance of CST for multiple processes associated with telomere replication and genome-wide replication rescue. They also strongly suggest that different DNA binding transactions are needed for CST to resolve different forms of replication stress with a subset of these transactions being disrupted by STN1-OBM. While STN1-OBM did not inhibit interactions with TPP1 or pol α (Fig 4), it is possible that STN1-OBM disrupts CST interaction with as yet unidentified partner proteins. If this is the case, the interaction of STN1 with such proteins might provide an additional mechanism to target CST to its various sites of action within the genome.

Our *in vitro* DNA binding studies using CST(WT) and CST(STN1-OBM) provide new insight into the mechanism of mammalian CST binding to ssDNA. Past studies provided conflicting information concerning the sequence specificity of CST binding [23,26]. We now confirm that human CST binds long (48 nt) substrates with little sequence specificity, however sequence identity is important for binding to short (18 nt) substrates as the telomeric G-strand substrate TelG-18 is bound with high affinity while binding to the non-telomeric substrate NonTel-18 is undetectable. We also provide evidence that human CST harbors multiple DNA binding domains. The STN1-OB fold comprises one of these domains and based on structure prediction, we suggest that OB folds in CTC1 comprise the others. Since CST only bound the 18 nt substrate that had the sequence of telomeric G-strand DNA (Fig 1), the domain(s) that bind short oligonucleotides (i.e. the STN1 OB fold or an adjacent OB fold in CTC1) must provide important determinants for sequence-specific binding. Given that long substrates (telomeric and non-telomeric) are bound with higher affinity than short substrates and their binding is less affected by STN1-OBM, it seems likely that these substrates contact additional DNA-binding domains beyond those used to contact short substrates.

The known structural similarity between STN1-TEN1 and RPA2-RPA3, together with the likely presence of multiple OB folds in CTC1, had previously suggested an RPA-like binding mechanism whereby mammalian CST contacts DNA via multiple OB folds. However, this was not a foregone conclusion because *S*. *cerevisae* CST binds DNA through one high affinity binding site in Cdc13 [49]. While our work supports the multiple OB fold binding mechanism for mammalian CST, it also reveals significant differences between CST and RPA in the contributions made by individual subunits during binding to ssDNA. For RPA, the only binding sites for short substrates correspond to the OB folds of RPA1 that bind proximal to the DNA 5’ end [41,42]. These OB folds also comprise the highest affinity binding sites. However, for CST, both CTC1 and STN1 contact short DNA substrates and STN1, which binds near the DNA 3’ end, is necessary to stabilize binding. These findings imply that the high affinity binding sites in CST are contributed by STN1 and CTC1 and they interact with DNA towards the 3’ end. While this architecture differs from that of RPA, it is well suited for CST to bind a telomeric 3’ overhang.

Despite the above differences between the two protein complexes, RPA can still be used as a model to help us understand the relationship between CST function and its mechanism of DNA binding. The ability of RPA to act as a hub that directs the sequential loading and unloading of partners such as Rad51 and Rad52 or SV40 T-antigen and pol α stems from the dynamic nature of RPA binding to ssDNA [41,44,63]. Because RPA utilizes 4 OB-folds to bind DNA, individual OB folds can undergo rapid microscopic dissociation and re-association from the DNA without causing the whole protein to dissociate [43,44]. Instead the rapid dissociation and re-association of individual OB folds is what enables RPA to diffuse along DNA to melt DNA structure or load and unload partner proteins [45].

Given that mammalian CST is likely to bind DNA via a similar number of OB-folds, it is possible that CST binding is also dynamic. If so, microscopic dissociation of individual OB folds from ssDNA could enable CST to engage or disengage interaction partners from the DNA (Fig 7E). Like the CST complex from *Candida glabrata*, mammalian CST might also be able to resolve unwanted DNA structure such as G quadruplexes (G4) [64]. The dynamic binding model for CST action is appealing because it can explain why CST is involved in multiple steps during telomere replication and in the resolution of diverse forms of replication stress. It can also explain many of the phenotypes of CST depletion. For example, during telomere replication, CST might aid in removal of G4 structure from the lagging strand during replication of the duplex DNA and it may engage pol α on the G-overhang to initiate C-strand fill-in following telomerase action. The role in G4 structure removal could explain why STN1 depletion leads to a slowing of telomere duplex replication with formation of MTS. Likewise, the role in pol α engagement could explain why C-strand fill-in is disrupted despite pol α remaining associated with the telomere [21,25]. The ability of CST to engage pol α to initiate DNA synthesis at dormant or late firing origins could also explain why STN1 depletion inhibits replication restart after genome-wide replication fork stalling. Moreover, resolution of DNA structure at G-rich or regions of repetitive sequence could underlie the role of CST in resolving endogenous replication stress at non telomeric loci [27,30,31].

While current models for CST function have focused on the regulation of DNA pol α, the large size of CTC1 suggests that CST will have many interaction partners. Thus, mammalian CST may well direct the actions of additional proteins involved in the resolution of replication stress. A broader understanding CST function will require the identification of these proteins and analysis of how CST modifies their ability to engage with stalled forks, replication origins or other replication intermediates.

If having multiple DNA binding domains and a dynamic DNA binding mechanism is so important for CST function in mammals, one has to ask why *S*. *cerevisiae* Cdc13 uses only one OB fold to bind DNA [49] and *S*. *pombe* appears to lack a Cdc13/CTC1 subunit [65]. One possibility is that the multiple DNA binding domains necessary for dynamic binding are provided through dimerization or alternative subunit stoichiometries such as those found *in S*. *cerevisiae* and *C*. *glabrata* [50,64]. An alternative answer could lie in the division of labor between CST and RPA and how this has evolved between organisms. In *S*. *pombe*, RPA cooperates with the helicase Pif1 to help resolve G4 structures at lagging strand telomeres [66,67] and a simple Stn1/Ten1 complex appears sufficient to regulate telomerase to pol α switching for C-strand fill-in [65,68]. Thus, a full CST complex with dynamic DNA binding properties may be unnecessary for telomere maintenance. Perhaps a CST complex is also superfluous for genome-wide replication rescue because *S*. *pombe* RPA has adapted to function in these processes.

## Materials and Methods

### Cell lines

HeLa 1.2.11 STN1 knockdown (clone shSTN1-7), shSTN1 rescue (STN1-Res), control non-target (clone shNT-3) and TEN1 overexpressing cell lines were as described previously [24,25,30]. To create the STN1-OBM cells, the three amino acid mutations (W89A, R139L and Y141A) were introduced by PCR mediated mutagenesis into the shRNA-resistant FLAG-STN1 allele previously used to make the shSTN1-Res cells [24]. shSTN1 cells were transfected with retrovirus encoding shRNA-resistant FLAG-tagged STN1-OBM and the Thy1.1 marker. Cells expressing STN1-OBM were isolated by FACS based on Thy1.1 expression and were re-sorted periodically to maintain expression.

#### Analysis of telomere phenotypes and DNA fiber analysis

Telomere FISH was performed using FITC-(TTAGGG)_3_ probe as previously described [24]. Genomic DNA was isolated using the Promega Wizard Kit and G-overhang abundance was determined by non-denaturing in-gel hybridization with ^32^P-labeled (AATCCC)_4_ probe. For the DNA fiber analysis, cells were labeled with IdU and CldU, DNA spread and fibers stained and visualized essentially as described [24]. At least 145 fibers and 5 images were scored for each independent experiment. See supplemental materials for more details.

#### Co-immunoprecipitation

To detect TPP1 interaction with STN1, HEK293T cells were transfected with HA-mCherry-TPP1 [57] and FLAG-STN1 (WT or OBM) expression constructs. To detect interaction between CST and DNA pol α, HEK293T cells were transfected with FLAG-CTC1, FLAG-STN1 (WT or OBM) and TEN1 expression constructs. Cells were extracted 72 hrs later with 20 mM Tris pH 8.0, 100 mM NaCl, 1 mM MgCl_2,_ 0.1% NP-40 (Igepal). TPP1 was precipitated with HA antibody, STN1 and CTC1 were precipitated with FLAG beads (Sigma A2220).

#### CST expression and purification

SF9 cells were co-infected with the baculovirus encoding FLAG-CTC1, His-STN1/STN1-OBM or TEN1 (all tags were N-terminal). Infected cells were lysed in 25 mM Tris-HCl pH 7.5, 500 mM NaCl, 0.5% NP-40, 1 mM PMSF and protease inhibitor cocktail. The supernatant was supplemented with 1 mM DTT and 25 mM imidazole and applied to nickel-sepharose beads (GE healthcare 17-5268-01). The beads were washed twice with 25 mM Tris pH 7.5, 500 mM NaCl, 0.5% NP40, 25 mM imidazole, 1 mM DTT and once with 25 mM Tris pH 7.5, 500 mM NaCl, 25 mM imidazole, 1mM DTT. The protein was eluted with 25 mM Tris 7.5, 500 mM NaCl, 100 mM imidazole, 10% glycerol, 1 mM DTT and then diluted 1:4 with 25 mM Tris pH 7.5, 175 mM NaCl, 10% glycerol, 1 mM DTT to bring the imidazole to 25 mM and NaCl to 300 mM. FLAG beads were added to the diluted protein and incubated for 1 hr at 4°C. Beads were washed with 25 mM Tris pH 7.5, 200 mM NaCl, 10% glycerol, 1mM DTT. The protein was eluted with 3X FLAG peptide (Sigma F4799) stored at 4°C. The concentration was determined by PAGE and silver staining using a BSA standard.

#### Electrophoretic mobility shift assays and UV crosslinking

CST(WT) or CST(STN1-OBM) (0.5–20 nM) was incubated with ^32^P-labeled oligonucleotide (0.1 nM) in 25 mM Tris pH 8.0, 1 mM DTT, 150 mM NaCl for 30 min at RT. For UV cross-linking 20 nM CST was incubated with 2 nM ^32^P-labeled oligonucleotide for 30 min and then subject to cross-linking in a Stratagene Hybridization Oven for 30 min on ice at full power (7200mJ/cm^2^) using 6 UVA bulbs.

#### Filter binding assay to determine Kd,app and t½

Double-filter binding assays were performed as described [69,70] using a 72-well minifold vacuum manifold slot blot apparatus and nitrocellulose and HyBond XL filters (GE Healthcare). Phosphorimager screens were scanned on a TyphoonTrio phosphorimager (GE Healthcare) and the amount of bound versus free DNA was quantified using ImageQuantTL software. Graphpad prism software was used to for plotting and curve fitting (one site specific saturation binding equation for Kd and one phase exponential decay equation for t½). To determine Kd,app, CST (0.01–20 nM) was incubated with ^32^P-labeled oligonucleotide (0.01 nM) in binding buffer (25 mM Tris pH 8.0, 1 mM DTT, 150 mM NaCl) for 18 hrs at 4°C to reach reaction equilibrium. To determine the dissociation rate (t½), 20 nM CST was incubated with 0.1 nM ^32^P-labeled oligonucleotide for 1 hr at 4°C. 0.5 μM of the corresponding unlabeled oligonucleotide was then added. Samples were analyzed by filter binding assay after 0, 0.5, 1, 3, 6, 9 or 18 hrs incubation with cold competitor.

#### Protein threading and structure prediction

CTC1 protein sequence was analyzed for structural homology to know proteins using Phyre2 and HHpred. The top hits identified with Phyre2 included homology between: (i) CTC1 a.a. 791–1109 and U. maydis RPA 70 a.a. 226–560 (prob. 98%). This region of RPA 70 encompasses OB folds B and C and part of OB A. (ii) CTC1 a.a. 219–408 and the OB folds domains from human POT1 a.a. 18–246 (prob. 85%). (iii) CTC1 a.a. 608–703 and the OB fold of human TPP1 a.a. 117–213 (prob. 79%). Homology was also found between the above regions of CTC and OB-fold containing regions of other proteins (e.g. SSB). Similar results were seen with HHpred. Based on these findings, we suggest that CTC1 harbors a total of 6 OB folds. The two in the N-terminus are most similar to the OB folds found in POT1, the three C-terminal OB folds resemble those of RPA70 while the central OB fold appears to resemble that of TPP1.

## Supporting Information

S1 Fig (A) PCR and sequencing strategy to monitor cells for presence of the wild type sh-resistant *STN1* allele versus *STN1-OBM*. The cartoon indicates relative location of exons in endogenous *STN1* mRNA. Arrowhead indicates exon with mutations. Arrows indicate locations of primers used for PCR (black) or sequencing (dotted). (B) Telomere FISH of metaphase spreads from shSTN1, shNT, STN1-Res and STN1-OBM cells. Representative images show that STN1-OBM does not cause increased telomere fusion or telomere loss. White arrows, MTS; green, FITC-(C_3_TA_2_)_3_ probe; blue, DAPI. (C) Non-denaturing in-gel hybridization showing telomeric restriction fragments from the indicated cell lines. Mean telomere lengths are shown at the bottom. Values represent the weight averaged mean from 3 or 4 independent experiments ± SD.
(TIF)

S2 Fig (A) FACS analysis showing cell synchronization of shSTN1, STN1-OBM and STN1-Res cells used to analyze G-overhang length. (B) Co-immunoprecipitation of DNA pol α with CST. Extracts were from cells transfected with the indicated constructs. CST was precipitated with FLAG beads, these were then heated to 50°C and loaded on the gel. Western blots were performed with antibody to Pol α, STN1, TEN1 or FLAG. The Western blots with STN1 and TEN1 antibody show only the overexpressed protein because the levels of endogenous protein are too low to detect with the exposures that are shown.
(TIF)

S3 Fig Quantification of tracks scored during DNA fiber analysis with the indicated cell lines. The table shows total number of tracks scored for each replication event. Number in brackets indicates the percent of total tracks.
(TIF)

S4 Fig (A) Representative slot blots used to determine DNA binding affinity (Kd) for CST(WT) and CST(STN1-OBM) binding to NonTel-36 or TelG-18. DNA concentrations are shown in brackets. (B) Representative slot blot used to determine t½ for CST(WT) and CST(STN1-OBM) binding to NonTel-36 or TelG-18. Time of incubation with cold competitor DNA is shown in brackets.
(TIF)

S5 Fig Photocrosslinking of CST subunits to unmodified or 3’ thiothymidine substituted TelG-18. CST(WT) or CST(STN1-OBM) was were incubated with unmodified or modified TelG-18, samples were irradiated with UVA, separated in SDS gels and analyzed by phosphorimaging. * indicates cross-linking products observed only in some experiments. Markers on the phosphorimager scans were obtained by laying the gels on nitrocellulose membrane and marking the positions of the marker bands with radioactive ink.
(TIF)

S1 Text Supplemental Materials and Methods. (DOC)
