# Supplementary figures and images for: STN1 OB Fold Mutation Alters DNA Binding and Affects Selective Aspects of CST Function
Source: PLoS Genet. 2016 Sep 30;12(9):e1006342. doi: 10.1371/journal.pgen.1006342 (PMC5045167; doi:10.1371/journal.pgen.1006342)

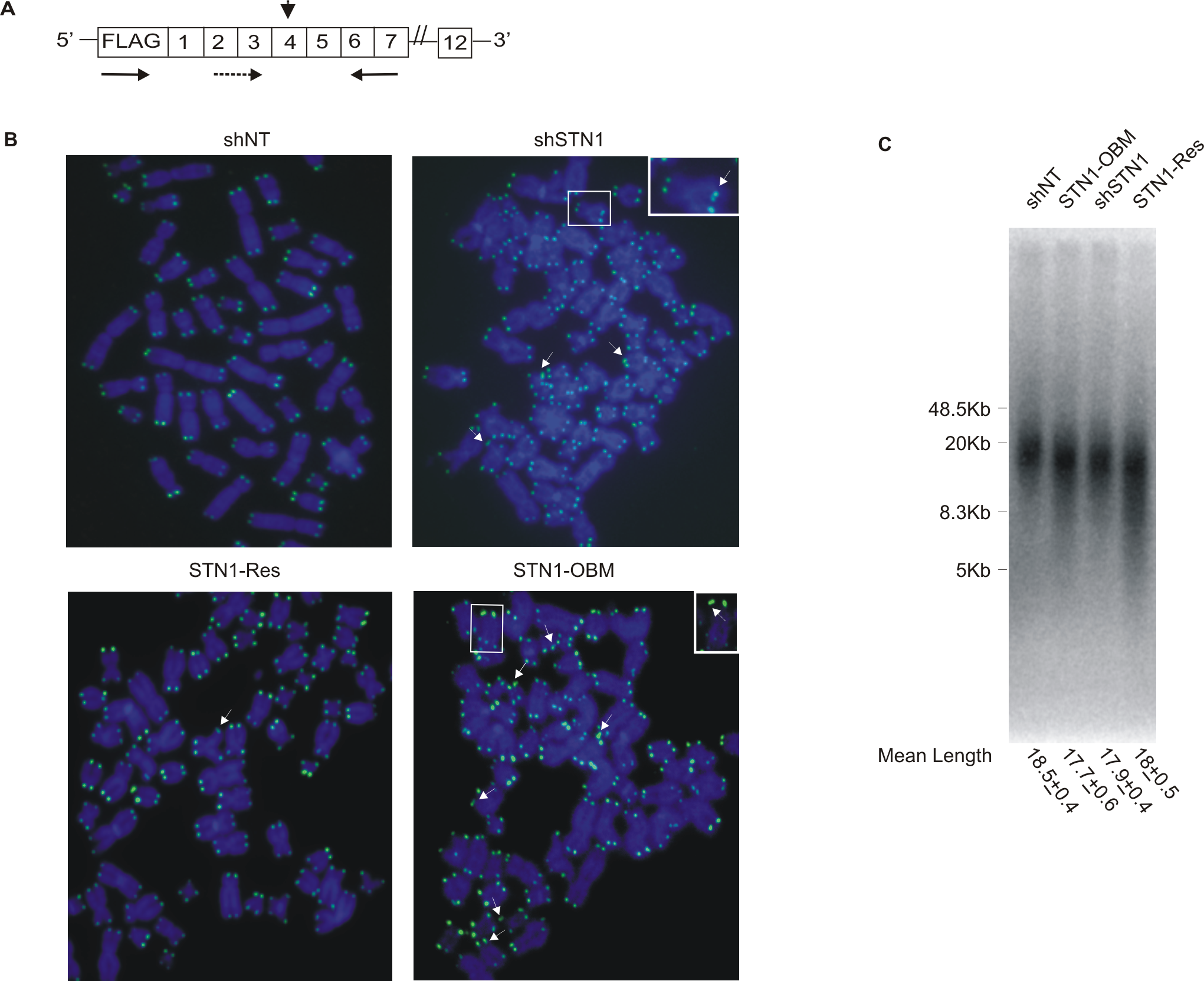

Supplement: S1 Fig — (A) PCR and sequencing strategy to monitor cells for presence of the wild type sh-resistant STN1 allele versus STN1-OBM. The cartoon indicates relative location of exons in endogenous STN1 mRNA. Arrowhead indicates exon with mutations. Arrows indicate locations of primers used for PCR (black) or sequencing (dotted). (B) Telomere FISH of metaphase spreads from shSTN1, shNT, STN1-Res and STN1-OBM cells. Representative images show that STN1-OBM does not cause increased telomere fusion or telomere loss. White arrows, MTS; green, FITC-(C3TA2)3 probe; blue, DAPI. (C) Non-denaturing in-gel hybridization showing telomeric restriction fragments from the indicated cell lines. Mean telomere lengths are shown at the bottom. Values represent the weight averaged mean from 3 or 4 independent experiments ± SD. (TIF) [file pgen.1006342.s001.tif]

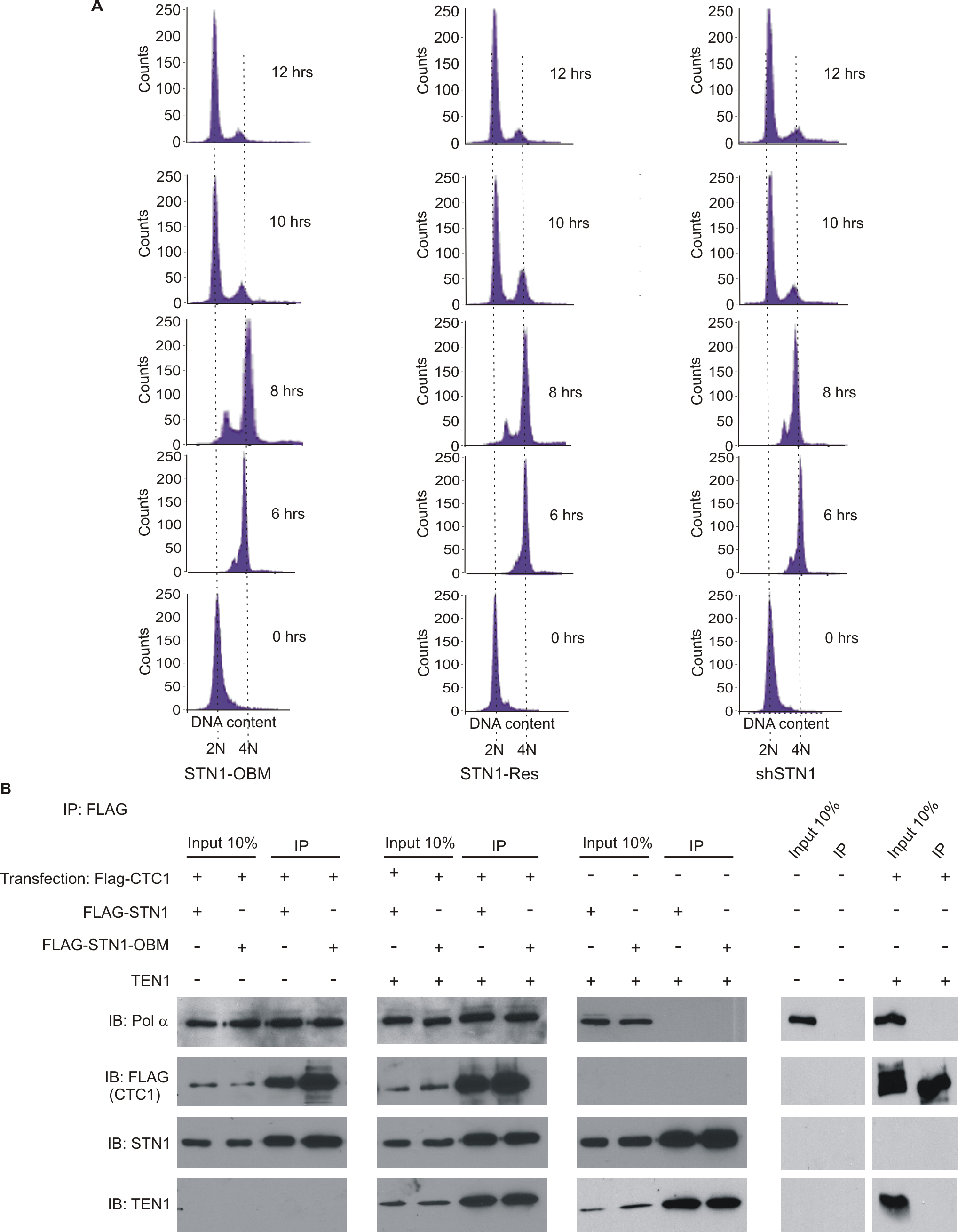

Supplement: S2 Fig — (A) FACS analysis showing cell synchronization of shSTN1, STN1-OBM and STN1-Res cells used to analyze G-overhang length. (B) Co-immunoprecipitation of DNA pol α with CST. Extracts were from cells transfected with the indicated constructs. CST was precipitated with FLAG beads, these were then heated to 50°C and loaded on the gel. Western blots were performed with antibody to Pol α, STN1, TEN1 or FLAG. The Western blots with STN1 and TEN1 antibody show only the overexpressed protein because the levels of endogenous protein are too low to detect with the exposures that are shown. (TIF) [file pgen.1006342.s002.tif]

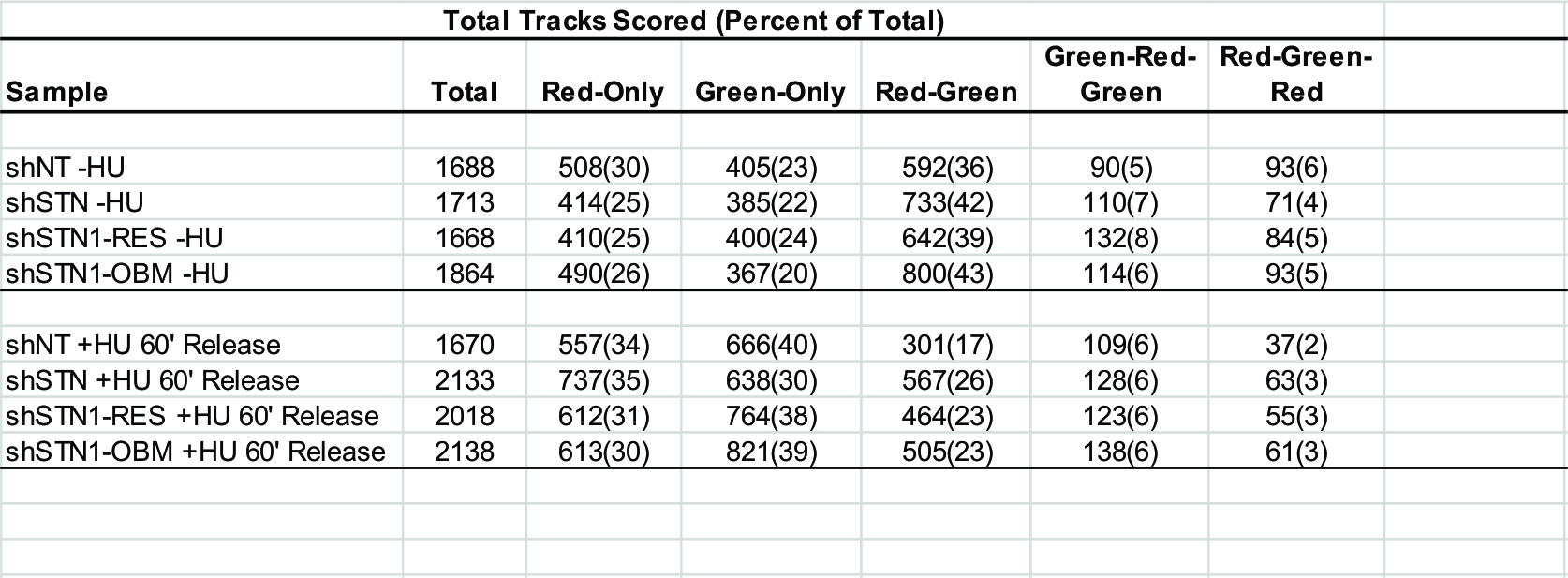

Supplement: S3 Fig — The table shows total number of tracks scored for each replication event. Number in brackets indicates the percent of total tracks. (TIF) [file pgen.1006342.s003.tif]

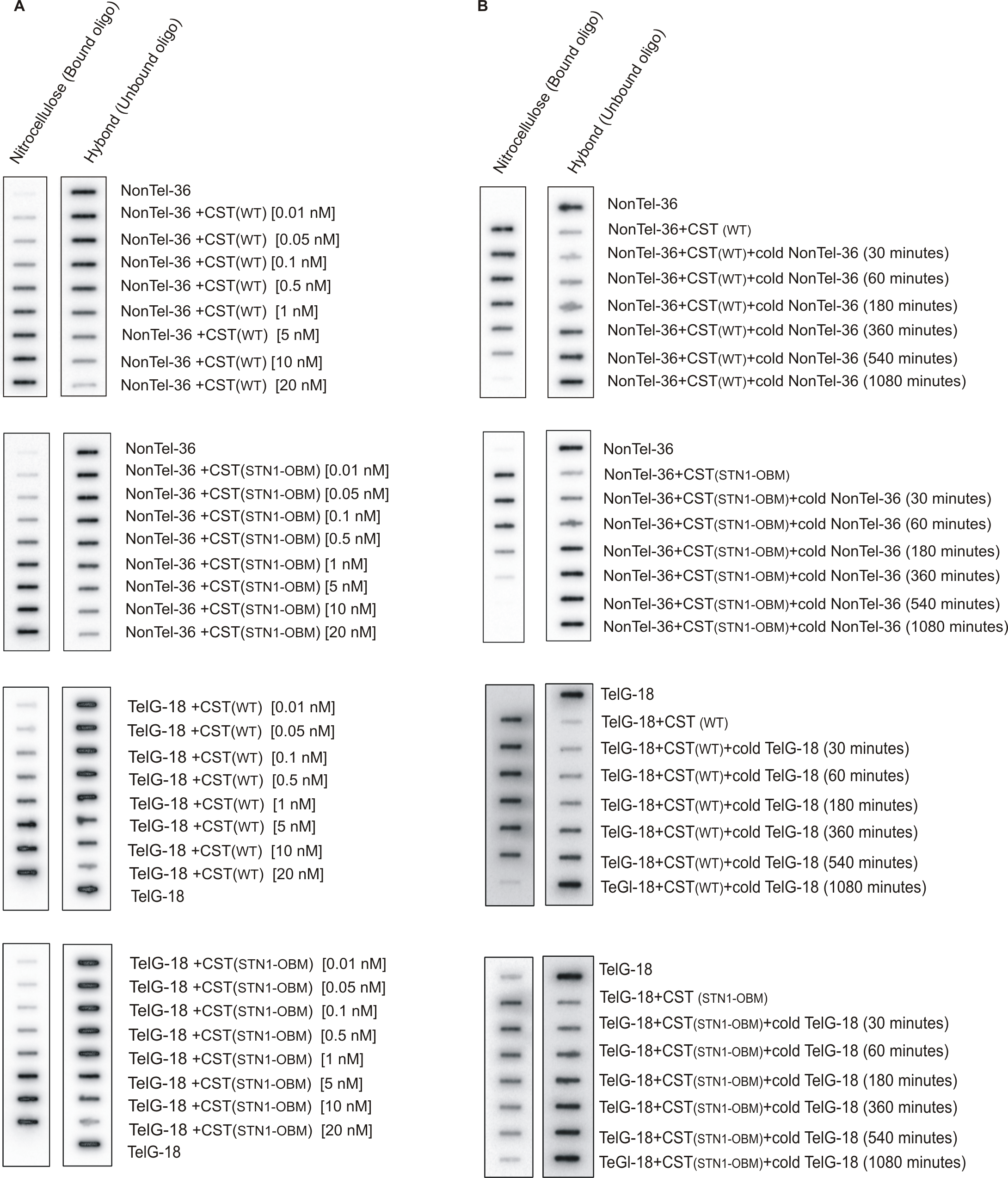

Supplement: S4 Fig — (A) Representative slot blots used to determine DNA binding affinity (Kd) for CST(WT) and CST(STN1-OBM) binding to NonTel-36 or TelG-18. DNA concentrations are shown in brackets. (B) Representative slot blot used to determine t½ for CST(WT) and CST(STN1-OBM) binding to NonTel-36 or TelG-18. Time of incubation with cold competitor DNA is shown in brackets. (TIF) [file pgen.1006342.s004.tif]

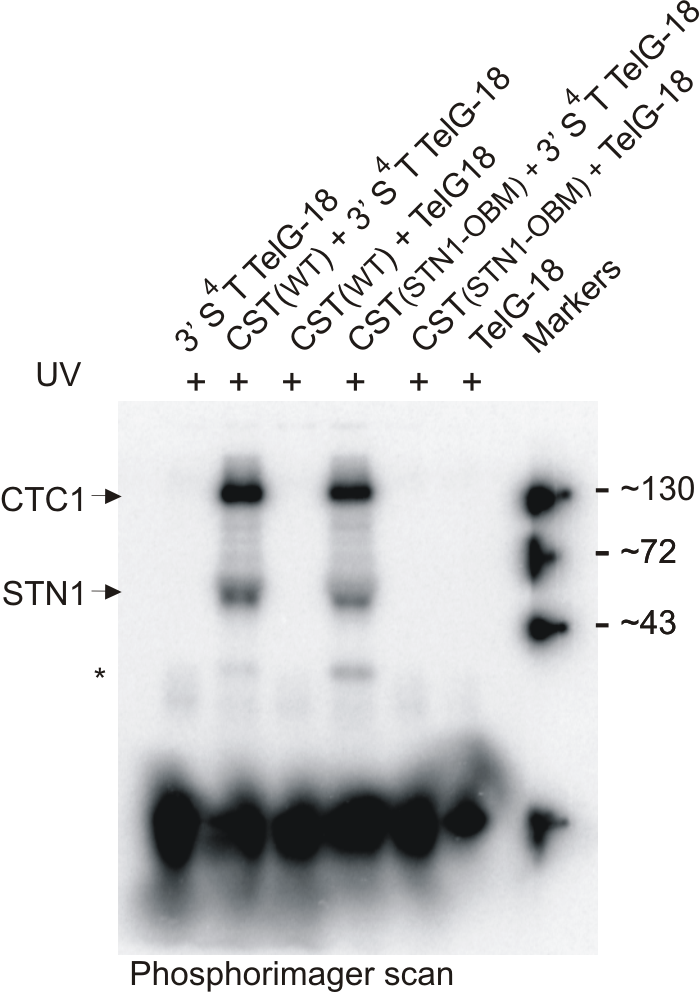

Supplement: S5 Fig — * indicates cross-linking products observed only in some experiments. Markers on the phosphorimager scans were obtained by laying the gels on nitrocellulose membrane and marking the positions of the marker bands with radioactive ink. (TIF) [file pgen.1006342.s005.tif]
