# Supplementary material for: STN1 OB Fold Mutation Alters DNA Binding and Affects Selective Aspects of CST Function
Source: PLoS Genet. 2016 Sep 30;12(9):e1006342. doi: 10.1371/journal.pgen.1006342 (PMC5045167; doi:10.1371/journal.pgen.1006342)
Supplement: S1 Text — (DOC) [file pgen.1006342.s006.doc]

**Supplemental Figures**

**Fig S1.** (A) PCR and sequencing strategy to monitor cells for presence of the wild type sh-resistant *STN1* allele versus *STN1-OBM*. The cartoon indicates relative location of exons in endogenous *STN1* mRNA. Arrowhead indicates exon with mutations. Arrows indicate locations of primers used for PCR (black) or sequencing (dotted). (B) Telomere FISH of metaphase spreads from shSTN1, shNT, STN1-Res and STN1-OBM cells. Representative images show that STN1-OBM does not cause increased telomere fusion or telomere loss. White arrows, MTS; green, FITC-(C_3_TA_2_)_3_ probe; blue, DAPI. (C) Non-denaturing in-gel hybridization showing telomeric restriction fragments from the indicated cell lines. Mean telomere lengths are shown at the bottom. Values represent the weight averaged mean from 3 or 4 independent experiments ± SD.

**Fig S2**. (A) FACS analysis showing cell synchronization of shSTN1, STN1-OBM and STN1-Res cells used to analyze G-overhang length. (B) Co-immunoprecipitation of DNA pol α with CST. Extracts were from cells transfected with the indicated constructs. CST was precipitated with FLAG beads, these were then heated to 50°C and loaded on the gel. Western blots were performed with antibody to Pol α, STN1, TEN1 or FLAG. The Western blots with STN1 and TEN1 antibody show only the overexpressed protein because the levels of endogenous protein are too low to detect with the exposures that are shown.

**Fig S3.** Quantification of tracks scored during DNA fiber analysis with the indicated cell lines. The table shows total number of tracks scored for each replication event. Number in brackets indicates the percent of total tracks.

**Fig S4.** (A) Representative slot blots used to determine DNA binding affinity (Kd) for CST(WT) and CST(STN1-OBM) binding to NonTel-36 or TelG-18. DNA concentrations are shown in brackets. (B) Representative slot blot used to determine t½ for CST(WT) and CST(STN1-OBM) binding to NonTel-36 or TelG-18. Time of incubation with cold competitor DNA is shown in brackets**.**

**Fig S5.** Photocrosslinking of CST subunits to unmodified or 3’ thiothymidine substituted TelG-18. CST(WT) or CST(STN1-OBM) was were incubated with unmodified or modified TelG-18, samples were irradiated with UV, separated in SDS gels and analyzed by phosphorimaging. * indicates cross-linking products observed only in some experiments. Markers on the phosphorimager scans were obtained by laying the gels on nitrocellulose membrane and marking the positions of the marker bands with radioactive ink.

**Supplemental Materials and Methods**

**Generation of STN1-OB cells and verification of cell lines**

HeLa 1.2.11 cells were cultured in RPMI-1640 with 10% FBS, antibiotics and glutamine. The STN1 OB fold mutant (STN1-OBM) was made by PCR-mediated site-directed mutagenesis of STN1 cDNA using the following primers (mutations are underlined): W89A, 5'-TGCATCTGC**GC**

**C**AAAAAGTTGAATACTGAGTCTGTATCAGC and 5’-CAACTTTTT**GGC**GCAGATGCAGTTTA

TAACTCCAGTGC; R139L and Y141A, 5’-TCC**T**CACA**GC**CAGAGAAGAGCGAGAGATTCATG

CCACC and 5’-CTCTTCTCTG**GC**TGTG**A**GGATACTGCCTCTGACTCGG. The resulting sh-RNA resistant STN1-OBM gene was cloned into pMSCV-IThy1-1 retroviral vector, upstream of IRES and the gene encoding the Thy1-1 cell surface protein. Retrovirus was produced and used to infect HeLa shSTN1-7 cells. Cells were harvested 48 hours after infection, incubated with APC-conjugated anti-Thy1-1 antibody (BD-Pharmingen) and sorted by FACS to isolate Thy1-1/STN1 expressing cells. Pools of sorted cells were expanded and tested for STN1 expression by Western blot. A PCR-based assay was used to monitor the integrity of the STN1-OBM and STN1-Res cell lines because the two cDNAs only differed in sequence at the site of the STN1-OBM point mutations (Appendix Fig. S1A). To avoid amplification and sequencing of the endogenous gene locus, genomic DNA was amplified with primers directed to the FLAG tag and the junction between exons 6 and 7 of the STN1 cDNA (5’-AGCTGGTACCATGGATTATAAAGATGATGATGATAAA

CAGCCTGGATCCAGCCG-3’ and 5’-CAGGGCGCCTGGATTGCT-3’) (Fig. E1A). The products were then sequenced to verify the presence or absence of the mutant allele. The sequencing primer hybridized to the junction between exons 2 and 3 (5’-GCCAGGTGCCAGGTGTAT-3’). The shSTN1 cells were monitored regularly for STN1 depletion by Western blotting with antibody to STN1.

**Western blots.** Proteins were separated by SDS-PAGE and transferred to nitrocellulose membrane. The membrane was blocked with 5% milk and incubated with antibodies to STN1 (1:2,000) made by immunizing rabbits with full-length purified STN1 , Actinin (1:10,000) from Santa Cruz (sc-17829), HA (1:2000) from Cell signaling (3724S), FLAG (1:2000) rabbit from Sigma (F7425), Myc (1:1000) from LifeTein (LT0421), purified TEN1 antibody (1:1000) , DNA polα (1:1000) goat polyclonal from Santa Cruz (sc-5920), Goat-α-Mouse-HRP from Thermo Scientific (32430), Goat-α-Rabbit-HRP from Thermo Scientific (32460) and Donkey- α-Goat-HRP from Santa Cruz (sc-2020).

**Anaphase bridge analysis**. HeLa cells were grown on coverslips overnight prior to addition of nocodazole (50 ng/ml). After 4 hr the cells were released into fresh media for 45-60 min then fixed in 3% formaldehyde. The coverslips were washed with PBS, dehydrated with cold ethanol (70%, 90%, 100%) and mounted with fluoro-gel (Electron Microscopy Sciences) and 0.2 μg/ml DAPI. The slides were viewed under 100x with a Nikon Eclipse E400 fluorescent microscope equipped with a Spot 2 digital camera (Diagnostic instruments Inc.). The number of anaphase cells with bridges out of 200 total anaphase cells was scored for each sample.

**Telomere FISH.** HeLa cells were grown overnight to 40-50% confluency. Colcemid (0.5 μg/ml) was added for 1.5 hr, the cells were harvested, fixed in methanol acetic acid and used to make metaphase spreads. Telomere FISH was performed essentially as described , using FITC-(TTAGGG)_3_ probe (Biosynthesis). To amplify the FISH signal the slides were blocked with PBG (0.5% BSA and 0.2% cold water fish gelatin in 1x PBS) for 20 min after the final hybridization step. Slides were then incubated with 6 μg/ml biotinylated anti-fluorescein for 1-2 hr at room temperature followed by 16 μg/ml fluorescein avidin (Vector laboratories) for 1 hr at 37°C in a humidified chamber. The slides were washed with PBS, dehydrated with ethanol (70%,90%,100%) and mounted with Fluro-gel (Electron Microscopy Sciences) with 0.5 μg/ml DAPI. Telomere FISH images were taken at 100x. MTS (Multi-telomere signals) were scored blindly. At least 200 chromosomes were analyzed per independent experiment.

**Genomic DNA isolation**. ~5x10^6^ cells were washed with PBS, lysed in 1 ml nuclei lysis buffer (Promega Wizard kit) and treated with RNAse (10 ng/ml) for 30 min at 37°C for 30 min. Samples were then digested with Proteinase K (10 ng/ml) for 4-5 hrs at 37°C, cooled on ice for 15 min prior to addition of 350 µl protein precipitation buffer (Promega Wizard kit). Samples were incubated on ice for a further 15 min then centrifuged at 13,000 rpm for 30 min. DNA was precipitated with isopropanol and resuspended in TE.

**Cell cycle synchronization and G-overhang analysis**. Cells were synchronized at the G1/S boundary by double thymidine block as previously described . Synchrony was monitored by FACS analysis of DNA content. Cells were released into fresh media after the second thymidine treatment and harvested 0, 6, 8, 10 and 12 hrs later for DNA isolation. To analyze G-overhang abundance, samples were digested or mock-digested overnight with Exo1, followed by restriction digestion with HinfI and MspI and separated briefly in 1% agarose gels to keep the telomeric restriction fragments in a tight band. Gels were dried and hybridized with (TA_2_C_3_)_4_ probe under non-denaturing conditions. The DNA was then denatured, and the gel was rehybridized with the same probe. Bands were quantified by PhosphorImager using Image Quant software. For each sample, band intensity from the native gel was normalized to that of the denatured gel to control for differences in loading. To assess changes in overhang abundance rather than internal ssDNA, the normalized band intensity of the Exo1 digested sample was subtracted from the mock digested counterpart. For experiments with asynchronous cells, the value for the shNT sample was set to 1 and the other samples were normalized to this value. For experiments with synchronized cells, the value for the 0 hr time point for each cell type was set to one and the samples from other time points were normalized to this value. The normalized signals from multiple experiments were then averaged to get the S.E.M. or max/min values.

**Telomere length analysis.** Genomic DNA was digested with HinfI and Msp1 and separated in 1% agarose by pulse-field electrophoresis. The gel was incubated in NaOH to denature the DNA and then incubated with ^32^P-labeled (TA_2_C_3_)_4_ probe. Alternatively, the DNA was transferred to Hybond membrane and then hybridized with the (TA_2_C_3_)_4_ probe. Signal was quantified by PhosphorImaging, and mean telomere length was determined by dividing each lane into 100 boxes using ImageQuant and applying the formula ΣSig/Σ(SigI/LI), where Sig is the sum of the signal from all 100 boxes, SigI is the signal in an individual box, and LI corresponds to the average length of the DNA in that box as determined using DNA markers and a standard curve .

**MTT assay.** Cell viability and proliferation were monitored using the tetrazolium based MTT colorimetric assay . Cells were grown overnight in 24 well plates to 60-70% confluency. The culture medium was then replaced with DMEM containing 1 mg/ml MTT and left for 40 min at 37^o^C. The medium was removed and cells were washed with PBS 3 times. DMSO was added to dissolve the formazan crystals and left for 15 min with shaking at room temperature. The reaction intensity was measured with a multi-well scanning spectrophotometer (Synergy MX, BioTek) at 570nm in triplicates in a 96-well plate.

**DNA-fiber analysis.** Cells were labeled with 50 uM IdU for 15 mins. Control cells were then labeled with 100 um CldU for 20 mins. The remaining cells were treated for 2 mM Hu for 2 hr. The HU was then removed and the cells labeled with 100 μm CldU for 60 min. Cells were harvested, lysed and spread on slides by hydrodynamic flow as described . Slides were incubated with mouse α‐BrdU (1:500, Becton Dickson) and rat α‐BrdU (1:500, Accurate Chemical) to detect IdU and CldU respectively. The signal was then amplified and visualized by incubation with AlexaFluor 594 rabbit α‐mouse (1:1000) and AlexaFluor 488 chicken α‐rat (1:750) (Invitrogen) secondary antibodies and AlexaFluor 594 goat α‐rabbit (1:1000) and AlexaFluor 488 (1:750) tertiary antibodies. Confocal images of DNA fibers were acquired using a Zeiss LSM710 microscope. The fibers were scored using previously described software .

**Supplemental References**

Chastain PD, 2nd, Heffernan TP, Nevis KR, Lin L, Kaufmann WK, Kaufman DG, Cordeiro-Stone M. 2006. Checkpoint regulation of replication dynamics in UV-irradiated human cells. *Cell Cycle* **5**: 2160-2167.

Harley CB, Futcher AB, Greider CW. 1990. Telomeres shorten during ageing of human fibroblasts. *Nature* **345**: 458-460.

Kasbek C, Wang F, Price CM. 2013. Human TEN1 maintains telomere integrity and functions in genome-wide replication restart. *J Biol Chem* **288**: 30139-30150.

Mosmann T. 1983. Rapid colorimetric assay for cellular growth and survival: application to proliferation and cytotoxicity assays. *Journal of immunological methods* **65**: 55-63.

Stewart JA, Wang F, Chaiken MF, Kasbek C, Chastain PD, 2nd, Wright WE, Price CM. 2012. Human CST promotes telomere duplex replication and general replication restart after fork stalling. *EMBO J* **31**: 3537-3549.

Wang F, Stewart JA, Kasbek C, Zhao Y, Wright WE, Price CM. 2012. Human CST has independent functions during telomere duplex replication and C-strand fill-in. *Cell reports* **2**: 1096-1103.

Wang F, Stewart, J., Price, C. M. 2014. Human CST abundance determines recovery from diverse forms of DNA damage and replication stress. *Cell Cycle* **13**: 3488-3498.
